# The lichen market place

**DOI:** 10.1111/nph.18130

**Published:** 2022-04-27

**Authors:** Ilse Kranner, Gregor Pichler, Martin Grube

**Affiliations:** ^1^ Department of Botany University of Innsbruck Sternwartestraße 15 6020 Innsbruck Austria; ^2^ Institute of Biology University of Graz Holteigasse 6 8010 Graz Austria

**Keywords:** desiccation tolerance, lichens, mycobiont, photobiont, symbiosis

## Abstract

This article is a Commentary on Spribille *et al*. (2022), **234**: 1566–1582.

Market places can look different, but they follow similar rules, with traders making investments to maximize market gains. Noë & Hammerstein (1994) popularized the idea of biological markets, where exchanges of resources and services among organisms can be analyzed in market terms. This approach is particularly useful for the interpretation of mutualistic associations of different species, where partners achieve a self‐sustaining agreement by giving away goods they easily produce in exchange for other goods provided by their partners. By summarizing the current knowledge of lichens, Spribille *et al*. (2022; pp. 1566–1582) in their Tansley review in this issue of *New Phytologist*, outline how lichens could fit the theory of biological markets, where sugar alcohols containing multiple hydroxyl groups, referred to as ‘polyols’ play prominent roles, also contemplating the relationship between genotype and phenotype, and calling for a paradigm change regarding the rewards of the symbiotic state for the photosynthetic symbionts – certainly an enjoyable read!

‘The “dual functionality” of polyols as carbon sources and compatible solutes … combined with the recently recognized habit of symbionts to occur in multiple symbioses, creates the conditions for a multiplayer market place of rewards and penalties that could drive symbiont selection and lichen diversification.’

Lichens expose themselves in their natural habitats to our naked eye. They emerge as macroscopic forms of microscopic fungi through their interactions with photosynthetic partners, and ‘lichen thallus’ is the proper name of this self‐supporting vegetative structure. Most, but not all, lichen‐forming fungi shelter algal/cyanobacterial partners beneath a protective tissue‐like peripheral cortex layer formed by branched and short fungal hyphae in a common polysaccharide matrix (Spribille *et al*., 2020). This ‘lichen glue’ is composed by the thickened outer cell walls of the fungi, which tightly hold the pseudoparenchymatic structure together. Similar conglutination of cell walls is otherwise known from fungal sporocarps or stromata, although the ability to form such pseudoparenchymatic structures in the vegetative stage seems to be a key innovation required for keeping control over the colony of photosynthetic microbial partners – green microalgae or cyanobacteria. In this way, lichens can emerge from the surfaces of rocks, bark, and other substrates (Fig. 1).

**Fig. 1:**
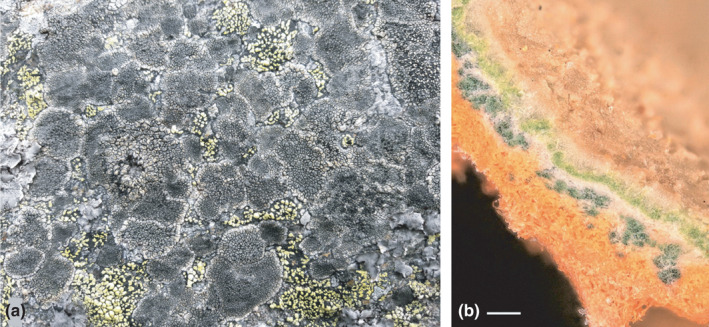
Lichen symbioses as biological markets. (a) The lichen market place on a rock surface, where different phenotypes of *Sporastatia testudinea* and *Rhizocarpon geographicum* compete for space. (b) Stratification of algal (upper green layer) and cyanobacterial (lower blue‐green layer) photosynthetic partners in the thallus of the chocolate chip lichen *Solorina crocea* (with typical orange lower side) suggests division of labor in nitrogen and carbon trading. Bar, (b) 300 µm.

However, lichen‐forming fungi are not the only fungi exposing themselves to the sunlight. A group of so‐called black fungi with strongly melanized cell walls commonly colonize surfaces where strong irradiation and periodic droughts prevail. Their cell‐wall melanins, which are crosslinked polyphenols, scatter the light and also cause structural rigidity to withstand mechanic forces from osmotic pressure variation (Gostinčar *et al*., 2009). Osmotic stress, to which these fungi are exposed similarly as are lichens, is also counteracted by the production of ‘osmoprotectants’ or ‘compatible solutes’ acting as osmolytes. Compatible solutes comprise various sugars and sugar alcohols, but also other low‐molecular‐weight molecules with neutral charge and low or no toxicity at high concentrations, including certain amino acids and their derivatives, for example mycosporines, and betaines. Efficient metabolism of osmolytes, as found in black fungi, was already suggested by Gostinčar *et al*. (2012) as a pre‐adaptation to facilitate the transition to a lichen symbiotic life style. They showed that black fungi can readily associate with green algae, but they do not enwrap algae in the same way as lichenized fungi do. Apparently the rigidity of strongly melanized cell walls does not favour the development of interspecific structures flexible enough to cope well with hygroscopic movements required for a poikilohydric life‐style. Lichens evolved another strategy to construct their thalli, with elastic cell walls glued together, maintaining integrity under changing hygric conditions in their natural habitat.

A key part of the Spribille *et al*. (2022) Tansley review is dedicated to previous work demonstrating that carbohydrates, including polyols, produced by the photobiont are exported to the fungal partner, which can convert them into other polyols. The authors highlight a ‘dual role’ of polyols, as substrates for growth and respiration, but also as important players in a lichens’ capability to survive desiccation. In turn, the photosynthetic symbiont would benefit from protection from herbivory and excess light provided by the fungus, while exerting leverage over fungal sex and morphogenesis (Spribille *et al*., 2022). In many lichens, shielding against strong light is mediated by crystallized fungal metabolites that accumulate in the intercellular spaces (sometimes termed ‘extrolites’) and may act as ‘smart switches’ letting more light pass to the photosynthetic powerhouse when crystals are more spaced in the hydrated state.

Lichens reboot full metabolic activity within minutes, with some proteins ready in place and not only available after resynthesis has re‐started via the ribosomal machinery. Lichens share this remarkable phenomenon of ‘desiccation tolerance’ with other life‐forms that have evolved to survive in the absence of water, such as other fungi, e.g. yeasts, as well as many bryophytes, a few ferns, a few hundred so‐called ‘resurrection angiosperms’ capable of surviving desiccation of their vegetative tissues, many desiccation tolerant seeds and pollen of higher plants, and a handful of specialized animals. ‘Anhydrobiosis,’ referring to the absence of water in ecological terms, is a somewhat imprecise term to describe desiccation tolerance, as on a cellular level, at least a few percent of dry mass comprise water bound to macromolecules. Desiccation tolerance can be defined as the ability to revive from the air‐dried state or, *sensu stricto*, as the capability of surviving drying at relative humidities below 65%, corresponding to a drop in absolute water contents to or below 0.1 g H_2_O g^−1^ dry mass and a water potential of ≤ −100 MPa (Oliver *et al*., 2020, and references cited therein). However, molecular mobility in the ‘dry’ state determining the (bio)chemical reactions that protect – or damage – cellular structures in the absence of water is still not fully understood. We know that molecular mobility is intricately linked with cellular water contents in conjunction with molecular composition. Enzymatic activity requires that at least the side groups of carbon backbones are able to move, which is the case in a ‘rubbery,’ but not a ‘glassy’ state, and more complex biochemical processes, including transcription and translation, entail transition to the liquid state with full mobility of the carbon backbone (Candotto Carniel *et al*., 2021; Farrant & Hilhorst, 2021). Unsurprisingly, the ability to withstand such low water potentials is based on more than one group of metabolites (Kranner *et al*., 2008; Oliver *et al*., 2020, and other topical reviews). Polyols appear to contribute to desiccation tolerance, but are certainly not the only factor responsible. Studies of other desiccation tolerant organisms failed to correlate the abundance of particular metabolites with desiccation tolerance. A famous example is trehalose, which has been suggested to confer desiccation tolerance, but this role has been disputed (Crowe *et al*., 2001) and a study with yeast mutants incapable of producing trehalose finally proved that trehalose is neither necessary nor sufficient for survival of desiccation (Ratnakumar & Tunnacliffe, 2006).

Whether the excess of algal carbon fixation mentioned by Spribille *et al*. (2022) remains largely segregated in soluble sugar and sugar alcohol pools that play a key role in desiccation tolerance, remains to be seen. What we do know is that they play important roles for the lichen symbiosis as forms of reduced carbon shunted between the symbionts. Interestingly, the kind of main algal polyols produced appears to reflect phylogenetic status of the photobiont. Tetritols, such as erythritol, appear to be the main goods offered by Chlorophyceae and Trentepohliales, whereas other clades including Prasiolales and Trebouxiales use pentitols, such as ribitol, and cyanobacteria trade with glucose (see citations and fig. 1 in Spribille *et al*. (2022)). Fungi convert glucose and ribitol into another pentitol, arabitol, and the hexitol mannitol, whereas erythritol appears to remain unchanged after import by the fungus.

Besides the well‐established involvement of compatible solutes in cellular protection from osmotic stress that accompanies desiccation, desiccation tolerance has been associated with many other mechanisms. Importantly, cellular structures need protection from molecular crowding that can lead to unwanted chemical reactions such as Maillard reactions. For rapid resurrection, key cellular structures and molecules such as nuceic acids, proteins, the cytoskeleton and organelles need to be protected upon drying and in the desiccated state, while retaining sufficient molecular order to revive upon rehydration. The involvement of chaperone‐like proteins (Xu *et al*., 2020), such as late embryogenesis abundant (LEA) proteins and LEA‐like proteins or ‘dehydrins,’ together with protection from oxidative stress, with key roles for thiol‐disulfide switches as central components of stress response (Zagorchev *et al*., 2013) are other important parts of the desiccation tolerance puzzle. Therefore, it appears that tolerance of the extreme abiotic stress factors that accompany a lichen’s everyday life is based on a combination of intricately interlinked biochemical and biophysical mechanisms. Lichens, sometimes called ‘extremophiles’ (Beckett *et al*., 2008) can survive desiccation and produce long‐lived thalli, representing a ‘song’ of co‐evolved functionality according to Doolittle & Inkpen (2018) ITNNTS (‘it’s the song, not the singer’) hypothesis, where variable symbionts could act as replaceable ‘singers’ in the market place of symbiotic interplay. Spribille *et al*. (2022) mention a range of additional microbial lichen‐associated partners of lichens. Besides the fungal–algal duet in the spotlight, their quiet background voices affords more attention to understand their roles in the slow‐growing lichen market places.
